# Answer – Abortion and Zika Virus Congenital Infection

**DOI:** 10.1055/s-0038-1668532

**Published:** 2018-08

**Authors:** Vivian Maria Ribeiro Mota, Alanna dos Santos Delfino, Thayse Elaine Costa Figueiredo Lopes, André Luiz Santos Pessoa, Erlane Marques Ribeiro, Luciano Pamplona de Góes Cavalcanti

**Affiliations:** 1 Universidade de Fortaleza, Fortaleza, CE, Brazil; 2 Universidade Federal do Ceará, Fortaleza, CE, Brazil; 3 Universidade Estadual do Ceará, Fortaleza, CE, Brazil; 4 Faculty of Medicine, Centro Universitário Christus, Fortaleza, CE, Brazil; 5 Hospital Infantil Albert Sabin, Fortaleza, CE, Brazil

We would like to thank for the comments of the letter to the editor about our article “Abortion in Cases of Zika Virus Congenital Infection.”^1^ Intrauterine consequences have become a focus of concern when it comes to Zika virus infection.^2^ As for the first statement: “There is still no evidence that Zika virus infection in pregnant women is the cause of abortion,” in fact, there are records in the scientific literature that strongly suggest this association and report intrauterine fetal death and spontaneous abortion,^3^ ^4^ both precocious^4^ and late,^2^ caused by the Zika virus and confirmed by serological tests on the fetus and the placenta.

We agree with our colleagues about the statement that not all cases of maternal infection by the Zika virus have as a result a fetus with microcephaly. This was initially demonstrated by a series of cases of babies born with normal cephalic perimeter, despite the confirmation of infection of the mother.^5^ In view of these findings, there is no doubt that the maternal infection by the Zika virus is not a reliable evidence of fetal infection. However, in our article,^1^ we present data that indicate that pregnant women throughout the world, due to the psychological distress and the epidemiology of fetal infection, may be deciding whether to undergo an induced abortion after being infected by the Zika virus Thus, we reinforce the statement that: “Early diagnosis may be useful for some purposes such as epidemiological monitoring, but should not be the presumptive data for the decision on abortion for the pregnant woman infected with zika virus.”

We believe that an early diagnosis provides the woman with a better understanding of the fetal situation, which empowers and enables her to decide what would be the best for her and the fetus, according to the law of each country.
